# Melanoma Associated Chitinase 3-Like 1 Promoted Endothelial Cell Activation and Immune Cell Recruitment

**DOI:** 10.3390/ijms22083912

**Published:** 2021-04-10

**Authors:** Gustavo Ramos-Espinosa, Yuanyuan Wang, Johanna M. Brandner, Stefan W. Schneider, Christian Gorzelanny

**Affiliations:** Department of Dermatology and Venerology, University Medical Center Hamburg-Eppendorf, Martinistraße 52, 20246 Hamburg, Germany; ramosespinosa@gmail.com (G.R.-E.); yuanyuan.wang@medma.uni-heidelberg.de (Y.W.); brandner@uke.de (J.M.B.); st.schneider@uke.de (S.W.S.)

**Keywords:** YKL-40, glycosaminoglycan, extracellular matrix, endothelium

## Abstract

Chitinase 3-like 1 (CHI3L1) is an enzymatically inactive mammalian chitinase that is associated with tumor inflammation. Previous research indicated that CHI3L1 is able to interact with different extracellular matrix components, such as heparan sulfate. In the present work, we investigated whether the interaction of CHI3L1 with the extracellular matrix of melanoma cells can trigger an inflammatory activation of endothelial cells. The analysis of the melanoma cell secretome indicated that CHI3L1 increases the abundance of various cytokines, such as CC-chemokine ligand 2 (CCL2), and growth factors, such as vascular endothelial growth factor A (VEGF-A). Using a solid-phase binding assay, we found that heparan sulfate-bound VEGF-A and CCL2 were displaced by recombinant CHI3L1 in a dose-dependent manner. Microfluidic experiments indicated that the CHI3L1 altered melanoma cell secretome promoted immune cell recruitment to the vascular endothelium. In line with the elevated VEGF-A levels, CHI3L1 was also able to promote angiogenesis through the release of extracellular matrix-bound pro-angiogenic factors. In conclusion, we showed that CHI3L1 is able to affect the tumor cell secretome, which in turn can regulate immune cell recruitment and blood vessel formation. Accordingly, our data suggest that the molecular targeting of CHI3L1 in the course of cancer immunotherapies can tune patients’ response and antitumoral inflammation.

## 1. Introduction

The tumor microenvironment is actively formed by a long list of actors [1]. Different cell types beyond cancer cells accumulate within the tumor tissue ranging from endothelial cells to infiltrating immune cells such as macrophages, neutrophils or T cells. Cytokines, chemokines and growth factors are essentially involved in cellular recruitment and propagation. The activity of these effector molecules is dose- and context-dependent and determines the fate of the tumor. Rebalancing of the tumor microenvironment by modern immune therapies promotes e.g., CD8^+^ T cell infiltration and thus tumor rejection [2]. Accumulation and deposition of most cytokines and growth factors are accomplished by the extracellular matrix within the tumor tissue and in particular by glycosaminoglycans such as heparin/heparan sulfate (HS) [3,4]. HS is a highly negatively charged polyelectrolyte exposed by proteoglycans, such as syndecans or perlecan [5]. Dysregulated HS turnover within the tumor tissue affects the composition of the tumor microenvironment and the recruitment of immune cells [6,7] and may also affect the efficiency of cancer immunotherapy [8]. The regulation of the extracellular matrix involves the action of a broad range of hydrolytic enzymes such as matrix metalloproteases or the HS degrading heparanase [6,9]. In the present manuscript, we postulate that mammalian chitinases also regulate the tumor microenvironment.

Mammalian chitinases belong to the glycosylhydrolase family 18 [10]. They can be further subdivided into enzymatically active enzymes capable of hydrolyzing chitin and enzymatically inactive enzymes often referred to as chitin lectins [10]. Members of the latter group lost their hydrolytic activity during evolution, but they were still able to bind chitin [11]. In humans, the expression of six chitinases has been documented. Next to the two active chitinases, chitotriosidase (CHIT1) and acidic mammalian chitinase (CHIA), four inactive chitinases are known, oviduct-specific glycoprotein (OVGP1), chitinase 3-like 1 (CHI3L1), chitinase 3-like 2 (CHI3L2) and chitinase domain-containing protein 1 (CHID1). The absence of chitin synthesis in mammals suggests that chitinases are involved in the defense of pathogenic fungi through the direct attack of chitin within the fungal cell walls [12,13,14]. However, upregulation of chitinase expression independent of infections suggests that chitinases have been re-educated during evolution to handle other physiological processes. For example, CHI3L1 also known as YKL-40 is elevated by chronic inflammation in the context of inflammatory bowel disease, rheumatoid arthritis or cancer. In melanoma, increased CHI3L1 serum levels were shown to correlate with disease severity and poor survival [15]. Within the melanoma tissue, CHI3L1 originates from the cancer cells and tumor-associated macrophages [16]. CHI3L1 may affect tumor development and metastasis by promoting angiogenesis, cell migration and by tuning the tumor microenvironment and tumor inflammation [17,18,19]. Beside its ability to bind chitin, CHI3L1 was shown to interact with type I collagen and HS [20,21]. Molecular dynamic simulations suggested that the CHI3L1 is also able to interact with hyaluronic acid [22]. These molecular relationships are most likely in close mechanistic connection to the biological activities of CHI3L1. Therefore, in the present work, we postulate that CHI3L1 release matrix-stored factors by competitive replacement. This soft matrix remodeling may contribute to the generation of chemotactic gradients within the tumor tissue and the fine-tuning of the tumor microenvironment.

## 2. Results

### 2.1. Comparative Expression Analysis of Mammalian Chitinases in Human Melanoma

**Figure 1:**
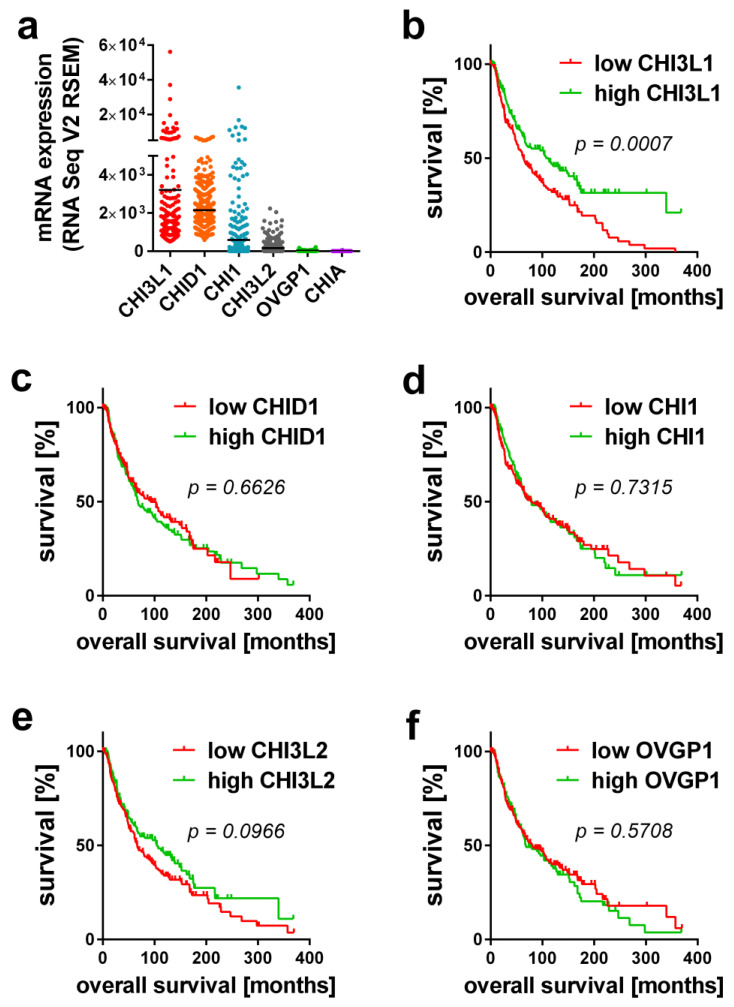
Analysis of public transcriptome data of melanoma patients. (**a**) Expression levels of mammalian chitinases in human melanoma tissue. (**b**–**f**) Kaplan-Meier curve presentation of the patients’ overall survival in correlation to the expression of (**b**) CHI3L1, (**c**) CHID1, (**d**) CHI1, (**e**) CHI3L2 and (**f**) OVGP1. Exact *p*-values of the performed log-rank test are indicated.

Prior to our experimental work, we searched the public transcriptome database cBioPortal to confirm the relevance of mammalian chitinases in melanoma tissues of more than 400 patients [23,24]. The analyzed data were part of the skin cutaneous melanoma TCGA PanCancer data. Gene expression is shown in relation to mRNA levels of all profiled samples and has been processed using RSEM (RNA-Sequencing by Expectation Maximization) [25]. Figure 1a shows the expression of all mammalian chitinases within the tumor tissue. CHI3L1 and CHID1 showed the highest expression, while the expression of CHI1 and CHI3L2 was only moderate. The expression levels of OVGP1 and CHIA were very low and, especially in the case of CHIA, mostly below the detection limit. Next, we correlated the expression of the chitinases with the patients’ survival (Figure 1b–f). In contrast to all other chitinases, only the expression of CHI3L1 correlated with a significantly increased survival (Figure 1b). The median survival in patients with low CHI3L1 expression was 63.3 months, while the median survival in patients with high CHI3L1 expression levels was 112.5 months. 

### 2.2. Biological Activity of CHI3L1 Is Related to the Melanoma Cell Glycocalyx

**Figure 2:**
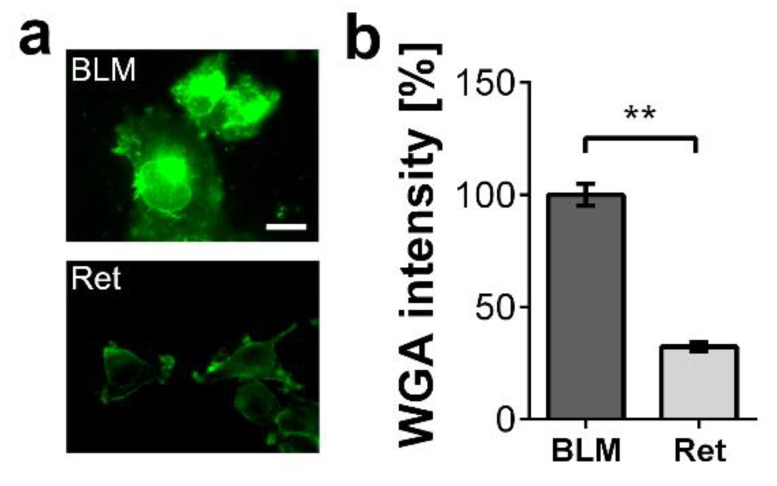
Glycosaminoglycan expression by the melanoma cell lines BLM and Ret. (**a**) Fluorescence microscopy of melanoma cell surfaces labeled with fluorescein isothiocyanate (FITC) conjugated WGA (green). Scale bar = 20 µm (**b**) Quantitative evaluation of the WGA staining intensity. *n* = 10 fields of view; ** = *p* ≤ 0.01 Student’s *t*-test.

We postulate that CHI3L1 modulates the tumor microenvironment by regulating the bioavailability of extracellular matrix-trapped cytokines or growth factors. In the first set of our experiments, we selected the two melanoma cell lines BLM (human) and Ret (murine) that produce different levels of glycosaminoglycans. We stained the surface of the two melanoma cells with wheat germ agglutinin (WGA). WGA detects N-acetyl glucosamine present in HS and hyaluronic acid, both components of the cancer cell glycocalyx and extracellular matrix [5]. In comparison to Ret cells, BLM cells bound significantly more WGA suggesting an elevated extracellular matrix production (Figure 2a,b). 

**Figure 3:**
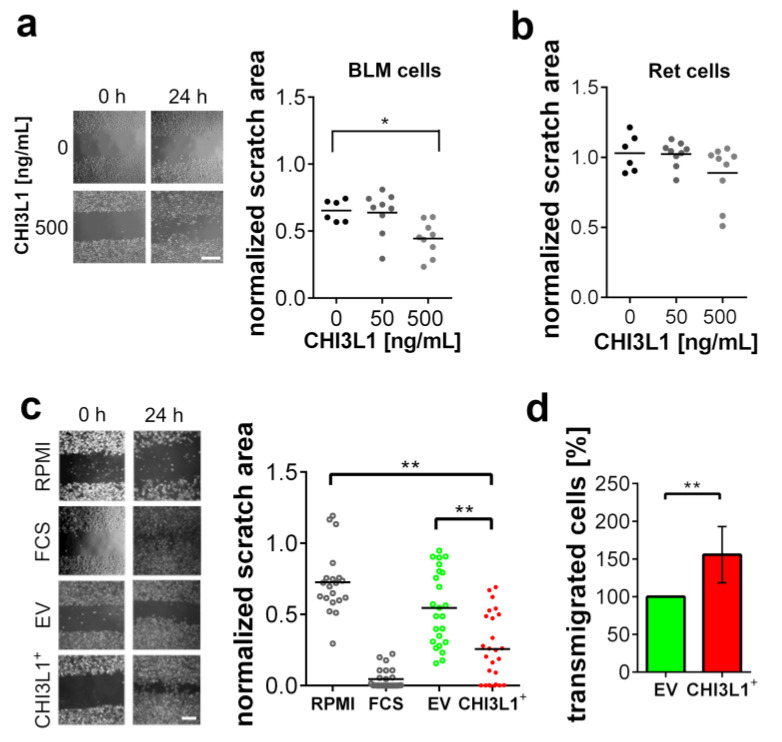
CHI3L1 promoted the migration of BLM cells and altered their secretome. (**a**) Representative bright-field microscopic images of the performed scratch assays with BLM cells. Treatment of BLM cells with 50 or 500 ng/mL CHI3L1 for 24 h. Scale bar = 100 µm. *n* ≥ 6; * = *p* ≤ 0.05 one-way ANOVA. (**b**) Treatment of Ret cells with 50 and 500 ng/mL CHI3L1 (**c**) Scratch assay with Ret cells treated with conditioned cell culture medium harvested from BLM cells. Supernatants from BLM cells overexpressing recombinant CHI3L1 (CHI3L1^+^) were compared to supernatants from empty vector-transfected BLM cells (EV). Ret cells cultivated in medium without FCS (RPMI) and Ret cells cultivated in medium containing 10% FCS (FCS) were used as experimental controls. Scale bar = 100 µm. *n* ≥ 20; ** = *p* ≤ 0.01 one-way ANOVA. (**d**) Transmigration assay analyzing the transmigration of Ret cells towards EV transfected BLM cells or CHI3L1 expressing BLM cells. *n* = 9; ** = *p* ≤ 0.01 Student’s *t*-test.

We treated both cell lines with different concentrations of recombinant CHI3L1 and measured their ability to migrate using scratch assays (Figure 3a,b). Ret cell migration was not affected by CHI3L1, while BLM cells showed an increased migratory potential upon treatment with 500 ng/mL recombinant CHI3L1. In further experiments, we overexpressed CHI3L1 in BLM cells and harvested the conditioned medium after 24 h. For the control, we used a conditioned medium harvested from BLM cells transfected with an empty vector (BLM EV). As shown in Figure 3c, Ret cells treated with conditioned medium from BLM cells overexpressing CHI3L1 (BLM CHI3L1^+^) showed a significantly increased migratory potential. Because scratch assays were not only sensitive to cell migration but also cell proliferation, we used transmigration assays as an additional experimental readout. To this end, we cultivated BLM EV or BLM CHI3L1^+^ cells in the lower cavity of the transwell chamber, while Ret cells were seeded into the upper cavity. Figure 3d indicate that significantly more Ret cells transmigrate towards the BLM CHI3L1^+^ cells than towards the EV control BLM cells. Taken together, our experiments suggest that CHI3L1 alone cannot induce Ret cell migration, whereas the conditioned medium of BLM CHI3L1^+^ promoted the migration of Ret cells significantly.

### 2.3. CHI3L1 Altered the Composition of the Tumor Cell Secretome

**Figure 4:**
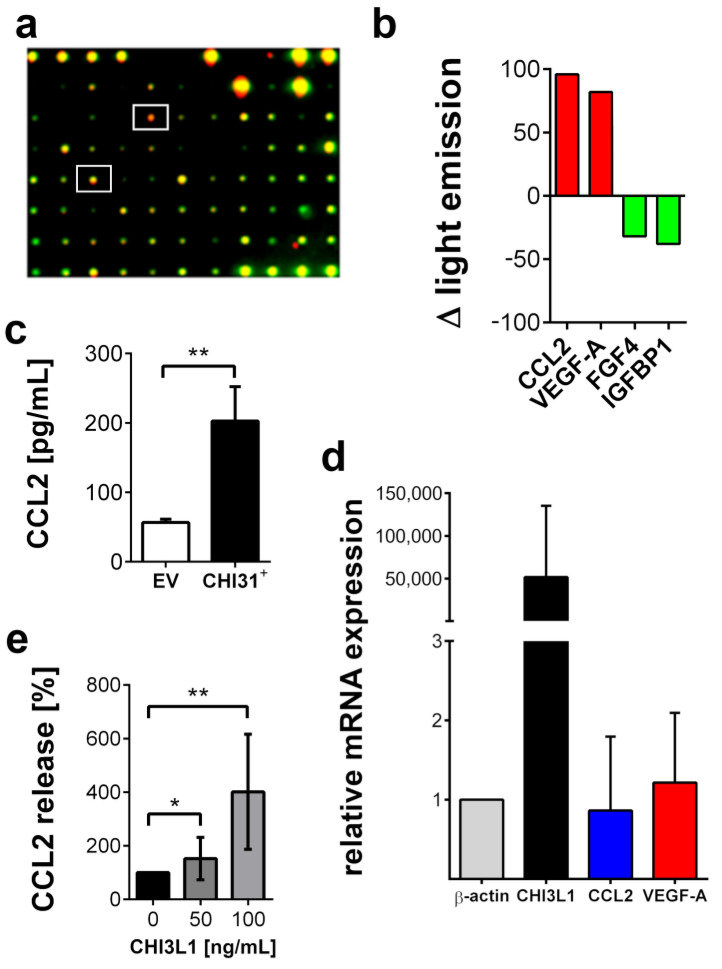
CHI3L1 affects the secretome of BLM cells. (**a**) Superimposed dot blot membranes probing the supernatants of BLM EV cells (green) and BLM CHI3L1^+^ cells (red). Red signals indicate proteins upregulated in BLM CHI3L1^+^ cells, green spots indicate proteins downregulated in BLM CHI3L1^+^ cells. White boxes mark the spots of CCL2 and VEGF-A. (**b**) Quantitative summary of the protein array. FGF4 = fibroblast growth factor 4. Additional data are presented in Supplementary Materials Figure S1. (**c**) CCL2 ELISA of supernatants obtained from BLM EV cells and BLM CHI3L1^+^ cells. *n* = 10; ** = *p* ≤ 0.01 Student’s *t*-test. (**d**) Expression analysis of CHI3L1, CCL2 and VEGF-A in BLM CHI3L1^+^ cells in comparison to BLM EV cells by qRT-PCR. Data were normalized to the expression of β-actin. The expression of CCL2 and VEGF-A was not significantly affected by CHI3L1 overexpression (**e**) BLM wild-type cells were treated with human recombinant CHI3L1 and CCL2 was measured in the supernatants by ELISA. *n* = 10; * = *p* ≤ 0.05, ** = *p* ≤ 0.01 one-way ANOVA.

In the next set of our experiments, we used a proteome microarray to compare the composition of the BLM CHI3L1+ conditioned medium with the conditioned medium of the BLM EV cells. CHI3L1 overexpression increased the levels of CCL2 and VEGF-A in the conditioned medium (Figure 4a,b). Independent ELISAs confirmed the significant increase in CCL2 (Figure 4c). Increased levels of CCL2 and VEGF-A were not coupled to an increased gene transcription as indicated by the qRT-PCR results shown in Figure 4d. These data suggest that CHI3L1 can increase the levels of growth factors and cytokines in the supernatants of melanoma cells independent of gene transcription. We have hypothesized that CHI3L1 can modulate the extracellular environment by replacing other HS-binding proteins such as growth factors or cytokines. To prove this notion, we treated BLM wild-type cells with 50 and 100 ng/mL of recombinant CHI3L1. In the supernatants of the treated cells, we analyzed the concentration of CCL2 by ELISA (Figure 4e). We found a CHI3L1 dose-dependent increase in CCL2 suggesting its liberation from the cell. 

**Figure 5:**
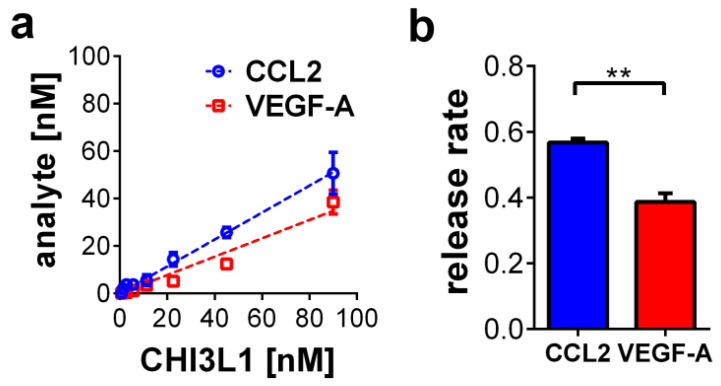
CHI3L1-mediated release of CCL2 and VEGF-A from HS. (**a**) CHI3L1 dose-dependent liberation of CCL2 and VEGF-A. *n* = 3–4 (**b**) Release rate of CCL2 and VEGF-A. *n* = 3–4; ** *p* ≤ 0.01 ANCOVA.

To further test whether CHI3L1 is able to liberate growth factors or cytokines from HS, we established a solid-phase binding assay (Figure 5). HS coated 96-well plates were loaded with recombinant VEGF-A or CCL2 and subsequently treated with different concentrations of CHI3L1 ranging from 0.7 to 90 nM. The amount of VEGF-A or CCL2 liberated from the HS coated 96-well plates was quantified by ELISA (Figure 5a). Both analytes were liberated from the plate by the addition of CHI3L1 in a dose-dependent manner. Figure 5b indicates that the release rate of CCL2 (0.6 ± 0.01) was significantly higher than the release rate of VEGF-A (0.4 ± 0.03; *p* < 0.01). 

### 2.4. CHI3L1 Promoted Immune Cell Recruitment and Angiogenesis

**Figure 6:**
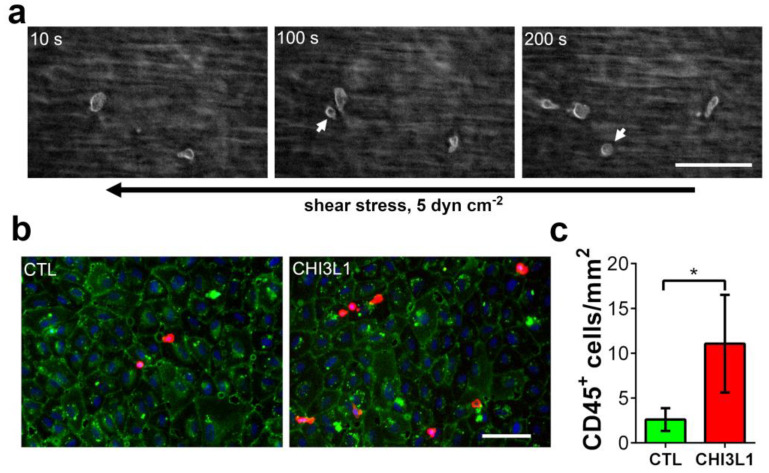
Microfluidic experiments indicate that CHI3L1 is able to promote adhesion of immune cells to endothelial cells. (**a**) HUVECs were perfused for 30 min with whole hirudin blood at a shear stress of 5 dyn cm^−2^. Shown are snapshots taken during the experiments by reflection interference contrast microscopy at indicated time points (10 s, 100 s and 200 s). The corresponding video is shown in Video S1. The black arrow indicates the flow direction. White arrows indicate leukocytes recruited from the flowing blood. Scale bar = 50 µm (**b**) Representative immune fluorescence images of the HUVEC layers after the flow experiment. HUVECs were stimulated with BLM cell supernatants, harvested from BLM cells either treated with 400 ng/mL CHI3L1 or PBS (CTL). CD31 was used as an endothelial cell marker (green); CD45 was used as an immune cell marker (red). Nuclei were stained with 4′,6-diamidino-2-phenylindole (DAPI, blue). Scale bar = 100 µm (**c**) Quantitative evaluation of immune cell adhesion. *n* = 5; * = *p* ≤ 0.05 Student’s *t*-test.

In further experiments, we investigated whether the ability of CHI3L1 to liberate cytokines, such as CCL2, from HS to promote the recruitment of immune cells to human umbilical vein endothelial cell (HUVECs). To this end, we stimulated HUVECs with the supernatants of BLM cells. The BLM cells have either been treated for 24 h with 400 ng/mL CHI3L1 or with vehicle control (phosphate-buffered saline, PBS). Subsequently, stimulated HUVEC cells were perfused with whole hirudin blood for 30 min at a shear stress of 5 dyn cm^−2^ using microfluidic channels. During the flow experiment, we imaged the adhesion of leukocytes to the endothelium by reflection interference contrast microscopy (Figure 6a). We found that leukocytes were continuously recruited to HUVECs, which were stimulated with supernatants harvested from CHI3L1-treated BLM cells. After the experiment, the adhesion of CD45+ cells was quantified by immune fluorescence microscopy (Figure 6b). As shown in Figure 6c, activation of HUVECs with supernatants from CHI3L1-treated BLM cells enhanced the adhesion of CD45^+^ immune cells to the endothelium, significantly. Upon treatment of HUVECs with BLM EV cell-conditioned medium, leukocyte adhesion remained at a low basal level [26]. 

**Figure 7:**
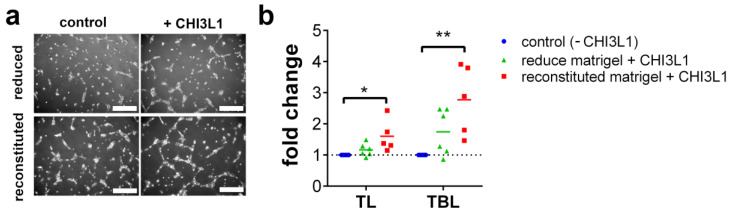
CHI3L1 liberated proangiogenic factors from matrigels promoted tube formation by HUVEC. (**a**) Representative images of HUVEC seeded on growth factor reduced or FCS reconstituted matrigel. HUVEC were either treated with PBS (control) or 400 ng/mL CHI3L1. (**b**) Automatized analysis of the total tube length (TL) and the total branching length (TBL). Data were normalized to the corresponding control experiment. *n* = 5; * *p* ≤ 0.05, ** *p* ≤ 0.01 one-way ANOVA.

The CHI3L1-mediated release of VEGF-A from HS suggests that CHI3L1 can indirectly promote angiogenesis. To prove this, we cultivated HUVECs on matrigels for 5 h and analyzed the total tube length and the total branching length. Moreover, to specifically address our hypothesis that CHI3L1 can liberate proangiogenic molecules from the extracellular matrix we compared growth factor reduced matrigels with matrigels reconstituted with 5% fetal calf serum (FCS) as a miscellaneous source of angiogenesis supporting factors. Figure 7a shows representative images of the tube formation assay. Quantitative analysis of the results is presented in Figure 7b. CHI3L1 treatment in combination with growth factor reduced matrigel increased tube formation, slightly. However, in comparison to the untreated controls (−CHI3L1) the increase in total tube length or the total branching length was not significant. In contrast, CHI3L1 treatment in combination with reconstituted matrigel enhanced tube formation, significantly. This result is in line with the data of the solid-phase binding assay indicating that CHI3L1 is able to promote tube formation through the liberation of proangiogenic factors from the extracellular matrix. 

**Figure 8:**
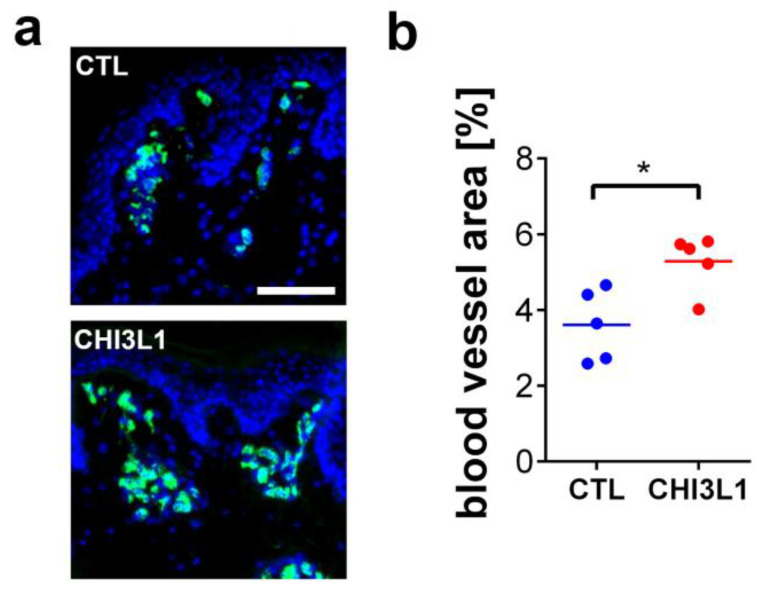
Treatment of porcine skin with 400 ng/mL recombinant CHI3L1 promoted angiogenesis ex vivo. (**a**) Representative immunofluorescence images of skin samples treated either with PBS (CTL) or CHI3L1. Nuclei were stained with DAPI (blue) and blood vessels were identified by staining of von Willebrand factor (green). Scale bar = 100 µm. (**b**) Quantitative analysis of the blood vessel area 48 h after treatment with CHI3L1. *n* = 5; * *p* ≤ 0.05 Student’s *t*-test.

To further support these data, we investigated the impact of CHI3L1 on neoangiogenesis in ex vivo skin samples. Punch biopsies of porcine skin were cultivated in an air-liquid interface configuration in which the dermis is placed in a culture medium, while the epidermis is exposed to the air [27]. After two days of cultivation, we quantified the number and size of dermal blood vessels by immunofluorescence detection of the blood vessel marker von Willebrand factor (Figure 8a). To analyze the impact of CHI3L1 on blood vessel formation, we added 400 ng/mL recombinant CHI3L1 into the culture medium. After treatment of the skin samples with CHI3L1, the area covered by blood vessels increased significantly in comparison to the vehicle-treated skin samples (Figure 8b).

## 3. Discussion

In the present study, we have shown that CHI3L1 affects the secretome of melanoma cells. Our data further indicate that the CHI3L1 induced alterations in the supernatants of melanoma cells are related to the competitive displacement of HS-bound molecules. CHI3L1 released factors promoted angiogenesis and immune cell adhesion to endothelial cells. In melanoma, the main source of CHI3L1 is tumor cells and tumor-associated macrophages [16]. The analysis of public transcriptome datasets also indicated that in comparison to other mammalian chitinases, expression levels of CHI3L1 were highest. Previously, high tumor burden and occurrence of metastasis in late-stage patients were mirrored by elevated CHI3L1 serum levels. Moreover, high CHI3L1 serum levels were shown to correlate with shorter overall survival [16]. Similar findings had been made for other cancer entities such as breast cancer, colorectal cancer or small cell lung cancer [28]. Our analysis of public transcriptome data indicates that elevated mRNA levels of CHI3L1 correlate significantly with increased overall survival. This different result might be related to the normalization of the provided mRNA sequencing data [25]. Expression levels are therefore independent of the total tumor burden within the patient. To further understand the role of CHI3L1 in melanoma and for the tumor microenvironment, we analyzed the secretome of human melanoma cells overexpressing CHI3L1. Proteome profiling microarrays showed the increased abundance of various cytokines and growth factors in the supernatant of BLM CHI3L1+ cells. We focused on CCL2 and VEGF-A as previous research suggested their molecular connection to CHI3L1 in the context of different cancers [29,30,31]. It has been shown that CHI3L1 can induce cell signaling and gene transcription by interaction with IL-13Rα2, CD44 or sydecan-1 [17,32,33]. To prove whether overexpression of CHI3L1 promotes the transcriptional elevation of CCL2 and VEGF-A in BLM cells we applied qRT-PCR. Our data indicate that neither CCL2 nor VEGF-A were transcriptionally regulated. Basing on this finding, we postulate that CCL2 and VEGF-A are liberated from the melanoma cell produced extracellular matrix. Indeed, the addition of recombinant CHI3L1 to wild-type BLM cells increases the levels of CCL2 within the cell supernatants. Moreover, our notion that CHI3L1 displace HS-bound cytokines is also supported by the findings from our solid-phase binding assay. In this assay, we mimicked the dose-dependent release of HS bound VEGF-A or CCL2 through the addition of recombinant CHI3L1. Interestingly, CCL2 was significantly better released from HS than VEGF-A suggesting a stronger molecular interaction between VEGF-A and HS. The binding of proteins to HS depends on the protein-specific HS-binding site and on the HS structure [3]. The high structural diversity of HS and the large range of different protein motifs capable to interact with HS impede straightforward mechanistic insight into the HS interactome. Our data suggest that CHI3L1 may tune the composition of the tumor microenvironment by the preferential release of specific cytokines or growth factors. However, to obtain a comprehensive picture of the CHI3L1-related alterations of the tumor microenvironment further research is required. To better understand the biological relevance of the competitive replacement of HS or matrix-bound factors by CHI3L1 we focused in our experiments on endothelial cell activation. Within the tumor tissue, the endothelium can be considered a gatekeeper that regulates the infiltration of immune cells and consequently the efficiency of immunotherapy [34,35]. To our knowledge, there are no clinical data available analyzing CHI3L1 in the context of current state-of-the-art immunotherapies applying CTLA-4 or PD-1 inhibition in melanoma. However, the mechanistic connection between CHI3L1 and immune checkpoint therapy has recently been documented in diffuse large B-cell lymphoma patients. Low response to anti-PD-1 monotherapy correlated with an increased CHI3L1 expression in immunosuppressive T cells [36]. In an experimental murine melanoma model, T cell-derived CHI3L1 was associated with enhanced lung metastasis and interferon-γ production [37] suggesting also a prominent role of CHI3L1 in melanoma immunotherapy. The repertoire of tumor-infiltrating immune cells is rather complex and a detailed analysis was beyond the scope of the present work [38]. However, in line with elevated CCL2 levels, we found increased recruitment of CD45^+^ leukocytes to endothelial cells, which had been stimulated with a conditioned medium harvested from CHI3L1-treated melanoma cells. Previous research suggested that CHI3L1 is associated with chemotaxis and migration of endothelial cells, smooth muscle cells and cancer cells [39,40]. We found that migration of Ret melanoma cells was promoted by the CHI3L1 enriched BLM conditioned medium but not by CHI3L1 alone. The derived concept of the CHI3L1-mediated soft matrix remodeling is also in good agreement with the results of our tube formation assay. CHI3L1 was able to promote tube formation of endothelial cells only in combination with matrigels reconstituted with proangiogenic factors. The proangiogenic activity of CHI3L1 was further confirmed in ex vivo skin samples. 

We cannot exclude additional CHI3L1-related actions within the extracellular matrix. Iwata et al. showed that CHI3L1 interferes with matrix metalloproteinase 1-mediated degradation of collagen type I [41]. Whether this interference is also related to competitive binding of CHI3L1 and the protease to collagen is to our knowledge not known but appears to be likely. Consequently, elevated CHI3L1 levels can prevent massive proteolytic matrix degradation, which in turn can preserve binding sites for matrix-bound factors. Interestingly, this may also explain our proteome profiling array data indicated that e.g., FGF4 and IGFBP1 levels were lower in supernatants of BLM CHI3L1^+^ cells.

In conclusion, understanding the high complexity and plasticity of the tumor microenvironment and the related interplay of contributing cells and molecules is challenging. Recent progress in immune checkpoint inhibition underpins the relevance of tumor inflammation and immune cell infiltration for cancer therapy outcome. The fundamental contribution of HS and HS-bound chemokines and growth factor for tumor inflammation is well known. However, to what extent the molecular structure of HS affects tumor development is elusive. In addition, knowledge on the bioavailability of HS-interacting molecules and their intrinsic competition for HS-binding sites within the tumor tissue is incomplete. In the present study, we showed that CHI3L1 can tune the tumor microenvironment by liberating HS-bound molecules such as CCL2 or VEGF-A. The related angiogenesis and immune cell recruitment indicate that CHI3L1 is involved in endothelial cell activation and tumor inflammation. Molecular targeting of CHI3L1 or regulating its activity within the tumor microenvironment may therefore represent a novel strategy to support current immunotherapies. 

## 4. Materials and Methods

### 4.1. Cell Culture

Ret cells [42] and BLM cells [43,44] were cultivated in RPMI-1640 medium (Capricorn Scientific, Ebsdorfergrund, Germany) supplemented with 1%L-Glutamine, 10% FCS and 1% Penicillin/Streptomycin (Sigma-Aldrich St. Louis, MO, USA). Ret cell medium contained additionally 1% non-essential amino acid solution (Sigma-Aldrich St. Louis, MO, USA). HUVECs were freshly isolated as previously reported [45] and cultivated in EGM-2 medium (Lonza, Basel, Switzerland). Human embryonic kidney cells 293 (HEK 293) cells were cultivated in DMEM (Capricorn Scientific, Ebsdorfergrund, Germany) supplemented with 1%L-Glutamine, 10% FCS and 1% Penicillin/Streptomycin or for production of recombinant CHI3L1 in Opti-MEM (Thermo Fisher Scientific, Waltham, MA, USA) supplemented with 1%L-Glutamine [46]. 

### 4.2. Expression of Recombinant CHI3L1

Human CHI3L1 cDNA generated from human macrophage-derived mRNA was cloned into the pDsRed-Monomer-N1 vector (Takara Bio Company, Mountain View, CA, USA) with a C-terminal 6xHis-tag. Stable transfected BLM and HEK293 cells were established through geneticin (800 µg/mL) selection (Thermo Fisher Scientific, Waltham, MA, USA). Expression of CHI3L1 was verified by qRT-PCR and Western blotting. Recombinant CHI3L1 was purified from HEK293 cell supernatants using HisTrap columns (GE Healthcare, Uppsala, Sweden). 

### 4.3. Proteome Profiling Microarray, Solid Phase Binding Assay and ELISA

The human Cytokine Array C5 (AAH-CYT-5, BioCat, Heidelberg, Germany) was used as described in the protocol provided by the manufacturer. To measure the CHI3L1-mediated release of VEGF-A or CCL2 from HS, we incubated 5000 ng/mL VEGF-A or CCL2 (R&D systems, Minneapolis, USA) for two hours in heparin-coated plates (Biotrend, Köln, Germany). After washing with PBS, wells were incubated for 2 h with recombinant CHI3L1 ranging from 3600 to 28 ng/mL and supernatants were collected. Subsequently, CCL2 and VEGF-A were detected in the supernatants by commercial ELISA kits (DuoSet^®^ CCL2 and VEGF-A, R&D Systems, Minneapolis, MN, USA). 

### 4.4. qRT-PCR 

qRT-PCR was performed as previously reported [47]. Briefly, Total RNA was extracted using the RNeasy Plus Mini Kit (Qiagen, Hilden, Germany) according to the manufacturer’s protocol and qRT-PCR was performed using the Reverse Transcription System and the GoTaq qPCR Master Mix (Promega, Heidelberg, Germany). The following primer pairs were used: VEGF-A: 5′-TCA CCA TGC AGA TTA TGC GGA-3′ and 5′-CTC CAG GGC ATT AGA CAG CA-3′; CCL2: 5′-CTC AGC CAG ATG CAA TCA ATG-3′ and 5′-GTT TGG GTT TGC TTG TCC AGG-3′; CHI3L1: 5′-AAG GCC TCT GTC GAC ATG GGT GTG AAG GCG TC-3′ and 5′-AGA ATT CGC AAG CTT CTA CGT TGC AGC GAG-3′. Expression levels were normalized to the endogenous β-actin gene (5′-CAT GTA CGT TGC TAT CCA GGC-3′ and 5′-CTC CTT AAT GTC ACG CAC GAT-3′). 

### 4.5. Fluorescence Microscopy 

Fluorescence microscopy was performed as previously reported [5]. Briefly, BLM and Ret cells were cultivated on glass coverslips for 24 h. Cells were fixated with 4% paraformaldehyde (Electron Microscopy Sciences, Hatfield, PA, USA), washed three times with PBS and blocked with 2% BSA (Sigma-Aldrich St. Louis, MO, USA) in PBS for 1 h. We stained the cells overnight at 4 °C with FITC-conjugated WGA (Thermo Fisher Scientific, Waltham, MA, USA) diluted 1:1000. Mounted samples were analyzed by fluorescence microscopy (Observer z1, Zeiss, Oberkochen, Germany). Images were analyzed with ImageJ v1.52. 

### 4.6. Scratch Assay 

Ret or BLM cells were cultured in 24 well plates until they were confluent. The cell layer was wounded with a 100 µL pipette tip and detached cell were removed by washing. Cells were treated as indicated and bright field images of the scratch were taken at defined time points (0 h and 24 h post wounding). Scratch area was calculated from the images using ImageJ v1.52. The relative scratch closure was obtained by dividing the scratch area at time point 24h by the scratch area at time point 0 h. 

### 4.7. Transmigration Assay 

BLM EV cells or BLM CHI3L1^+^ cells (100,000 cells/well) were seeded into 24 well plates and cultivated until they reached confluency. Prior to the transmigration experiment, culture medium was replaced by serum-free RPMI 1640 medium (Capricorn Scientific, Ebsdorfergrund, Germany) and filter inserts with a pore diameter of 8.0 µm (Greiner Bio-One ThinCert™, Thermo Fisher Scientific, Waltham, MA, USA) were inserted. Calcein green (Thermo Fisher Scientific, Waltham, MA, USA) labelled Ret cells (30,000 cells/filter) were placed into the upper cavity of the filter. The number of Ret cells transmigrated to the bottom of the 24 well plate were counted after 24 h of co-culture by fluorescence microscopy (Observer z1, Zeiss, Oberkochen, Germany). 

### 4.8. Tube Formation Assay 

HUVECs (10,000/well) were seeded on growth factor reduced matrigel (Corning, New York, NY, USA), which was placed into angiogenesis µ-slides (ibidi, Munich, Germany). Prior to the seeding, HUVEC were kept at starvation conditions in EBM-2 medium (Lonza, Basel, Switzerland) for 7 h. Where indicated, the matrigel was reconstituted with 5% FCS. HUVECs were treated with EBM-2 medium with or without recombinant CHI3L1 (400 ng/mL). Tube formation was imaged after 5 h by bright field microscopy and images were analyzed with ImageJ v1.52 using the angiogenesis analyzer plugin. 

### 4.9. Microfluidic Experiments 

Microfluidic experiments were performed as previously described [9,26]. Briefly, 300,000 HUVECs were cultured on gelatin-coated µ-slides 0.2 Luer (IBIDI GmbH, Munich, Germany) for 48 h under slight flow (1 dyn cm^−2^). Where indicated, slides were pre-incubated for 7 h with BLM cell supernatant harvested from BLM cells that were treated with 400 ng/mL CHI3L1 or remained untreated. Microfluidic channels were perfused with hirudinated whole blood at a shear stress of 5 dyn cm^−2^ for 30 min. Adhesion of leukocytes to the HUVECs was detected in real time by reflection interference contrast microscopy (Observer.Z1, Zeiss, Oberkochen, Germany). After the experiment, slides were fixated with 4% (*v*/*v*) paraformaldehyde (Electron Microscopy Science, Hatfield, PA, USA) in PBS. Antibodies directed against CD31 (mouse anti-human monoclonal antibody, Agilent Dako, Santa Clara, CA, USA, dilution 1:150) as endothelial marker and CD45 (anti-human Allophycocyanin-conjugated antibody, Miltenyi Biotec, Bergisch Gladbach, Germany, dilution 1:100) as leukocyte marker. Secondary antibodies conjugated to Alexa Fluor 488 and 647 (ThermoFisher Scientific, Waltham, USA) were diluted in PBS containing 1%BSA at dilutions of 1:200 and 1:1000. DAPI was used to label nuclei. Slides were imaged with a 20x and 40x oil objective mounted to an Observer Z.1 (Zeiss, Oberkochen, Germany). Data were processed with Zen software (1.1.2.0, Zeiss, Oberkochen, Germany) and ImageJ v1.52.

### 4.10. Ex Vivo Angiogenesis Model

Punch biopsies of porcine skin were generated as previously reported [27]. Skin samples with a diameter of 6 mm were placed dermis down on gauze in 12 well plates filled with DMEM medium (Capricorn Scientific, Ebsdorfergrund, Germany) supplemented with 2% FCS, hydocortisone and penicillin/streptomycin (Sigma-Aldrich St. Louis, MO, USA) and were indicated with 400 ng/mL CHI3L1. The epidermis of the skin remained exposed to air (air-liquid interface configuration) and was incubated at 37 °C for 48 h in a humidified atmosphere containing 10% CO_2_. After the experiment, samples were snap-frozen in isopentane (Sigma-Aldrich St. Louis, MO, USA) precooled with liquid nitrogen and stored at −80 °C. Tissue sections were analyzed by immune histology as previously reported [48].

### 4.11. Statistics

Results are expressed as means ± SD of at least three independent experiments. Statistical significance was proven for multiple comparisons with one-way ANOVA followed by post-hoc Tukey correction or for pairwise comparison with unpaired, two-tailed Student’s *t*-test. ANCOVA was used to compare linear regressions. Calculations were conducted with GraphPad Prism (GraphPad Software, San Diego, CA, USA) v7.03. (* *p* ≤ 0.05; ** *p* ≤ 0.01).

## Data Availability

Original data can be obtained from the authors upon request.

## Supplementary Materials

The following are available online at https://www.mdpi.com/article/10.3390/ijms22083912/s1, Figure S1: Comparative secretome profiling of BLM EV and BLM CHI3L1^+^ cells, Video S1: Reflection interference contrast microscopy of HUVECs perfused with hirudin blood.
