# Supplementary material for: Melanoma Associated Chitinase 3-Like 1 Promoted Endothelial Cell Activation and Immune Cell Recruitment
Source: Int J Mol Sci. 2021 Apr 10;22(8):3912. doi: 10.3390/ijms22083912 (PMC8069096; doi:10.3390/ijms22083912)
Supplement: Supplementary file 1 [file ijms-22-03912-s001.zip › Ramos et al Supplementary Materials.pdf]

Supplementary Materials for

Melanoma Associated Chitinase 3-Like 1 Promoted Endothelial Cell Activation and Immune Cell Recruitment

Gustavo Ramos-Espinosa †, Yuanyuan Wang †, Johanna M. Brandner, Stefan W. Schneider and Christian Gorzelanny *

Department of Dermatology and Venerology, University Medical Center Hamburg-Eppendorf, Martinistraße 52, 20246 Hamburg, Germany; ramosespinosa@gmail.com (G.R.-E.); yuanyuan.wang@medma.uni-heidelberg.de (Y.W.); brandner@uke.de (J.M.B.); st.schneider@uke.de (S.W.S.)

* Correspondence: c.gorzelanny@uke.de; Tel.: +49-40-7410-58976

† These authors contributed equally to this work.

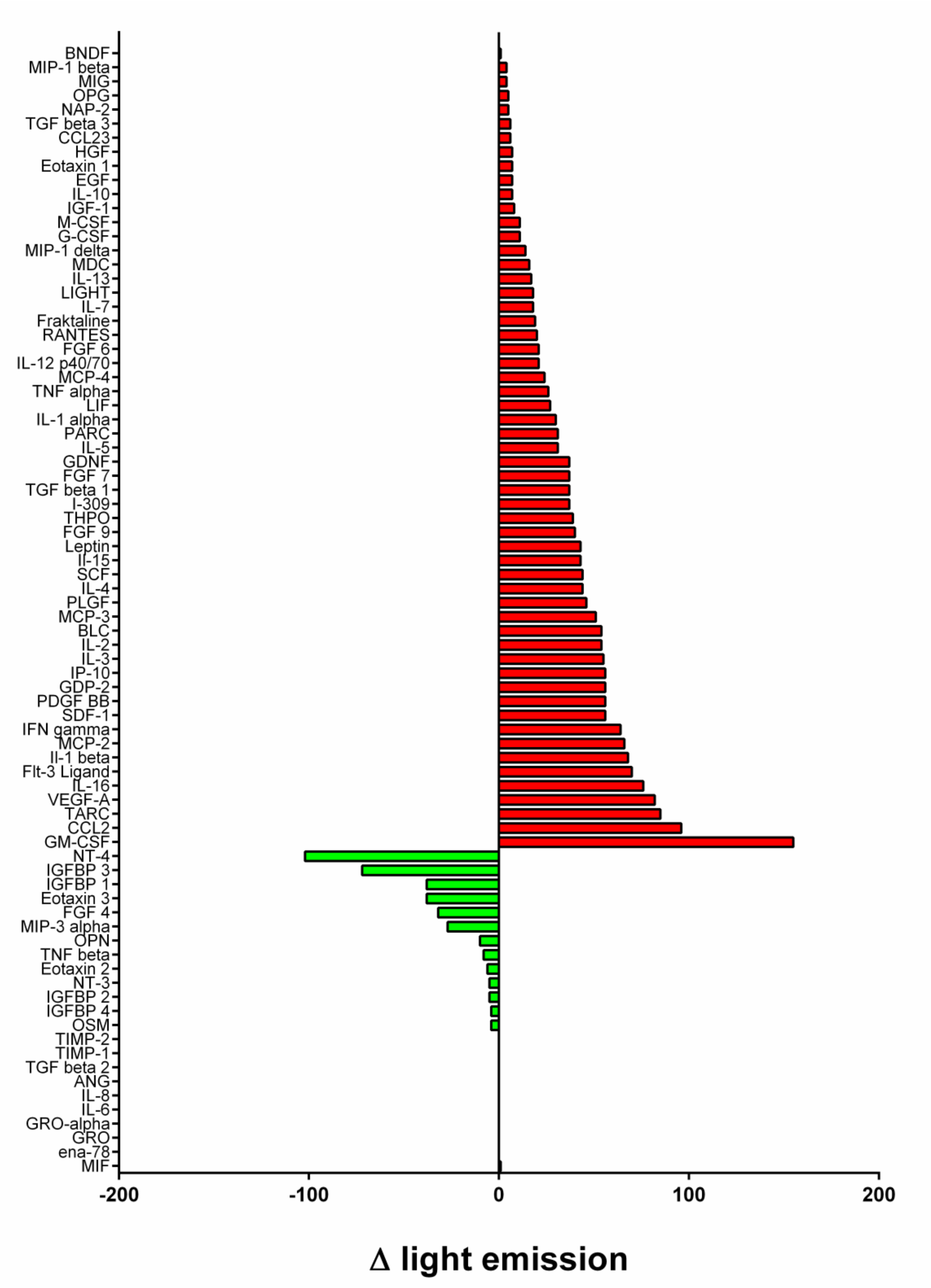

Figure S1. Comparative secretome profiling of BLM EV and BLM CHI3L1⁺ cells. ENA-78: epithelial-derived neutrophil-activating peptide 78 (CXCL5); GCSF: granulocyte colony-stimulating factor; GM-CSF: granulocyte-macrophage colony-stimulating factor; I-309: CCL1; MCP: monocyte chemoattractant protein; M-CSF: macrophage colony-stimulating factor; MDC: macrophage derived chemokine (CCL22); MIG: monokine induced by gamma interferon (CXCL9), MIP: macrophage inflammatory protein; RANTES (CCL5); SCF: stem cell factor; SDF: stromal cell-derived factor; TARC (CCL17); TGF: transforming growth factor; TNF: tumor necrosis factor; EGF: epidermal growth factor; IGF insulin-like growth factor; PDGF: platelet derived growth factor; BDNF: brain-derived neurotrophic factor; BLC: B lymphocyte chemoattractant (CXCL13); Ck beta 8-1: CCL23; Flt-3: Fms-related tyrosine kinase 3; GCP-2: granulocyte chemotactic protein (CXCL6); GDNF: glial cell-derived neurotrophic factor, HGF:

hepatocyte growth factor; IGFBP: insulin-like growth factor binding protein; IL: interleukin; IP-10: interferon gamma-induced protein 10 (CXCL10); LIF: leukemia inhibitory factor; Light: tumor necrosis factor superfamily member 14 (TNFSF14); MIF: macrophage migration inhibitory factor; NAP-2: neutrophil activating peptide (CXCL7), NT: neurotrophin; PARC: pulmonary and activation-regulated chemokine (CCL18), PLGF: placental growth factor, TIMP: tissue inhibitor of metalloproteinases.

Video S1. Reflection interference contrast microscopy of HUVECs perfused with hirudin blood. The continuous flow of blood was unidirectional and the applied shear stress was 5 dyn/cm^2. The frame interval during the experiment was 10 s. The frame rate in the video was set to 10 frames/s. The frame size corresponds to 156 x 98 μm.
